# The relationship between nocturnal hypoxia and sofa score on critically ill patients at the icu

**DOI:** 10.1186/2197-425X-3-S1-A764

**Published:** 2015-10-01

**Authors:** B Gucyetmez, HK Atalan, UA Turan, E Ozden, M Berktas, N Cakar

**Affiliations:** Acibadem International Hospital, Intensive Care Unit, Istanbul, Turkey; Atasehir Memorial Hospital, Intensive Care Unit, Istanbul, Turkey; Acibadem Kadıköy Hospital, Intensive Care Unit, Istanbul, Turkey; Antalya Memorial Hospital, Intensive Care Unit, Antalya, Turkey; Yeditepe University, PEPIRC, Istanbul, Turkey; Department of Anesthesiology and Intensive Care, Acibadem University Medical Faculty, Istanbul, Turkey

## Introduction

Apnea test (AT) (ApneaLink™, RESMED-Munich, Germany) can be used for different clinical conditions to calculate apnea-hypopnea index (AHI) and nocturnal hypoxia (NH) at intensive care unit (ICU)([1]). It's shown that obstructive sleep apnea syndrome (OSAS) and NH is associated post-operative complications, longer length of hospital stay, atrial fibrillation, pulmonary hypertension and nocturnal death([2, 3]). NH can lead to tissue hypoxia and thus a higher SOFA score at the ICU admission.

## Objectives

The purpose of the present study is to investigate the relationship between SOFA score at the ICU admission and NH.

## Methods

Upon the approval of local ethical committee, patients were screened prospectively and critically ill patients at the ICU older than 18 years were included whereas those with chronic obstructive pulmonary disease, lung cancer and OSAS were excluded. Of the 98 eligible ones; 10 patients with SOFA score ≤2 at the ICU admission and 10 with SOFA score>2 at the ICU admission were randomly selected regarding a priori sample size calculation. AT was performed for all patients at night before hospital discharge. Patient's age, gender, body mass index (BMI), charlson comorbidity index (CCI), lenght of time of AT, AHI, minimum SpO_2_ (min-SpO_2_), lenght of time for SpO_2_≤90%, percentage of nocturnal hypoxia (NH%) in the AT, minimum and maximum heart rate (min-HR and max-HR), APACHE II and SOFA scores and length of ICU and hospital stay were recorded. Groups were compared by using Mann Whitney U test due to non-normal distribution pattern.

## Results

Groups were similar in terms of age, gender, length of time for AT and min-HR (p>0.05 for each). Median BMI (27.7 kg/m2 vs. 25.5 kg/m2), APACHE II score (20.0 vs. 12.5), SOFA score (4.0 vs. 1.5), Charlson comorbidity index (5.0 vs. 4.0), AHI (33.0 vs. 20.5), length of time for SpO_2_≤90% (178.0 mins vs. 36.0 mins), NH % (51.2% vs. 13.6%), max-HR (133.0 bpm vs. 100.0 bpm), length of ICU stay (5.0 days vs. 3.0 days), length of hospital stay (5.0 days vs 2.5 days) of patients with SOFA score >2 were significantly higher than those with SOFA score ≤2 (p < 0.005 for each).

## Conclusions

Nocturnal hypoxia means the decrease in tissue oxygen delivery during sleeping. Undiagnosed OSAS and nocturnal hypoxia can cause an increase in CCI and SOFA scores at the ICU admission. According to our results, we can suggest that the patients who have a SOFA score higher than 2 at the ICU admission should be evaluated in terms of nocturnal hypoxia.
